# GLM7 – A Novel Composite Glycolipid Index Derived from Routine Health Indicators for Enhanced Diagnosis and Prediction of Multimorbidity

**DOI:** 10.1002/advs.202510552

**Published:** 2025-08-28

**Authors:** Zhihua Wang, Shuo Chen, Xiaojun Feng, Xi Chen, Paul C Evans, Hans Strijdom, Yu Ding, Jianping Weng, Suowen Xu

**Affiliations:** ^1^ Department of Endocrinology Centre for Leading Medicine and Advanced Technologies of IHM The First Affiliated Hospital of USTC Division of Life Sciences and Medicine University of Science and Technology of China Hefei 230001 China; ^2^ Anhui Provincial Key Laboratory of Metabolic Health and Panvascular Diseases Hefei 230001 China; ^3^ William Harvey Research Institute Barts and The London Faculty of Medicine and Dentistry Queen Mary University of London London EC1M6BQ United Kingdom; ^4^ Centre for Cardio‐metabolic Research in Africa Division of Medical Physiology ,Faculty of Medicine and Health Sciences, Stellenbosch University Cape Town 8000 South Africa

**Keywords:** disease diagnosis prediction, GLM7, machine learning, routine examination indicators

## Abstract

Routine health examinations for healthy adults typically involve measurements such as height, weight, blood biochemistry, complete blood count, and urinalysis. However, the current scope of physical examinations has expanded to include numerous tests, some of which have questionable insight into underlying pathology. In this study, we analyzed 26,289 samples from the NHANES (National Health and Nutrition Examination Survey) database, along with 49 included indicators, to systematically explore the correlation between conventional indicators and various diseases. Our aim was to establish new diagnostic and predictive indicators. Initially, the top 10 diagnostic and predictive indicators for five disease categories, namely cardiovascular diseases, diabetes, liver diseases, cancer, and comorbidities, are identified, and the reliability of the routine test indicators is emphasized. Moreover, GLM7 (glycolipid metabolism 7 factors), a novel indicator integrating seven routine factors, has been developed. Restricted cubic spline (RCS) analysis and forest plot evaluations reveal its relationships and risk thresholds across diseases. An extreme gradient boosting (XGBoost) model using these factors exhibits excellent predictive performance in both the NHANES discovery and CHARLS (China Health and Retirement Longitudinal Study) validation cohorts. This study confirms conventional indicators' efficacy and introduces GLM7 as a tool for disease diagnosis/prediction, providing new insights into precise disease management.

## Graphical abstract

This study leverages NHANES data to analyze 49 routine indicators, identifying the top 10 diagnostic markers for five major diseases and introducing GLM7, a novel indicator. Restricted cubic spline analyses, forest plot evaluations, and XGBoost modeling demonstrate conventional indicators' efficacy and GLM7's value for disease diagnosis/prediction.

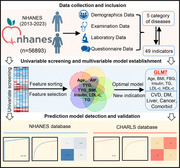

## 1 Introduction

Diagnostic testing has consistently accounted for a substantial proportion of healthcare expenditures, with some hospitals even adopting a “testing‐first” approach before clinical diagnosis.^[^ ^1^, ^2^ ^]^ The ever‐increasing number of diverse examinations not only consumes significant time and resources but also imposes a heavy economic burden on patients.^[^ ^3^, ^4^ ^]^ Routine health examinations serve as the cornerstone of population health maintenance, enabling the prompt identification of emerging health risks and the implementation of timely therapeutic measures.^[^ ^5^, ^6^ ^]^ For healthy adults, these examinations typically involve fundamental measurements such as height, body weight, blood biochemistry, blood count, and urinalysis. These tests have long been regarded as essential screening tools, offering critical insights into an individual's comprehensive health profile. However, the current landscape of physical examinations has evolved to include a wide array of tests, many of which may be unnecessary, raising concerns about the efficiency and accuracy of these assessments.^[^ ^7^, ^8^ ^]^

The importance of routine health examinations in disease prevention and early diagnosis cannot be overstated. By systematically evaluating various physiological parameters, healthcare professionals can identify deviations from normal ranges that may signify the onset of diseases. For instance, abnormalities in blood pressure, cholesterol levels, or blood glucose concentrations have been closely linked to the development of cardiovascular diseases (CVD) and diabetes mellitus (DM).^[^ ^9^, ^10^ ^]^ Similarly, liver function tests and markers of inflammation have proven useful in the early detection of liver diseases.^[^ ^11^ ^]^ Additionally, numerous novel indicators derived from routine testing parameters have emerged in recent years. For example, derivative indices such as triglyceride‐glucose index (TYG), triglyceride‐glucose‐body mass index (TYG_BMI), and atherogenic index of plasma (AIP) have been widely reported for their roles in predicting CVD and cardio‐renal metabolic syndrome;^[^ ^12^, ^13^, ^14^ ^]^ Red cell distribution width (RAR) shows utility in predicting inflammatory diseases;^[^ ^13^ ^]^ and uric acid to high‐density lipoprotein cholesterol ratio (UHR) has been implicated in the prediction of metabolic dysfunction‐associated steatotic liver disease (MASLD) and other disorders.^[^ ^15^ ^]^ These traditional testing indicators and newly derived markers have significantly advanced preventive and precision medicine. However, the challenge lies in distinguishing between truly informative indicators and those that contribute minimally to the diagnostic process, thereby avoiding unnecessary costs and potential patient discomfort.

Against this backdrop, the need to optimize routine health examinations becomes evident. The existence of numerous tests in standard physical examinations has led to a situation where the signal‐to‐noise ratio is compromised.^[^ ^16^ ^]^ This not only increases the burden on healthcare systems but also may dilute the focus on the most relevant indicators. As a result, there is a pressing need to re‐evaluate the utility of various routine testing indicators and their relationships with diseases, with the aim to establishing more targeted and efficient diagnostic and predictive tools.

This study endeavors to fill this gap by exploring the intricate relationships between various routine testing indicators and diseases. Specifically, we seek to identify the most informative indicators for the diagnosis and prediction of diverse diseases, with the ultimate goal of establishing novel indicators that can enhance the precision of disease management. By leveraging large‐scale epidemiological data and advanced statistical and machine learning techniques, we strive to provide new insights into the role of routine health examinations in modern healthcare.

## 2 Results

### 2.1 Baseline Characteristics of the Participants

**Figure 1:**
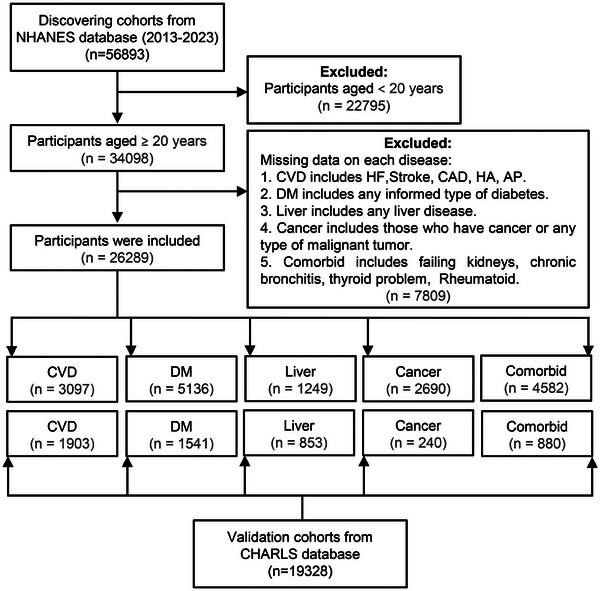
Flowchart of the sample selection from NHANES 2013–2023 and CHARLS 2011–2020. CAD: coronary artery disease; HA: heart attack; AP: angina pectoris; HF: heart failure; DM: diabetes mellitus; CVD: cardiovascular disease. NHANES: National health and nutrition examination survey; CHARLS: China health and retirement longitudinal study.

**Figure** **1** presents the flowchart outlining the sample selection procedure for this study. Following the application of pre‐defined inclusion and exclusion criteria, a final analytical sample comprising 26289 participants was established. The age distribution of the participants was as follows: 40.2% fell within the 20–44 years age bracket, 34.8% were aged 45–64 years, and 25% were 65 years or older. In terms of gender composition, 48.3% of the participants were male. Regarding educational qualifications, 8.92% had not completed high school, 91.1% possessed a high school diploma or had educational attainments beyond high school. Ethnically, the sample consisted of 13.5% Mexican American, 10.4% Other Hispanic, 36.1% Non‐Hispanic White, 23.3% Non‐Hispanic Black, and 16.7% participants classified under Other Race categories. With respect to marital status, 58.5% of the participants were married. In terms of lifestyle behaviors, 18.9% reported have history of heavy alcohol consumption, 57.7% were nonsmokers, and 56.6% engaged in minimal to no physical activity. Among the entire participant pool, 3097 (11.8%) were diagnosed with CVD, 5136 (19.5%) had DM, 1249 (4.75%) suffered from liver disease, 2690 (10.2%) had cancer, and 4582 (17.4%) presented with comorbidities including failing kidneys, chronic bronchitis, thyroid problems, and rheumatoid disorders. The baseline characteristics of the participants are comprehensively summarized in **Table** **1**. All observed disparities were found to be statistically significant (*p* < 0.05).

**Table 1:** Baseline characteristics of individuals.

		GLM7‐1	GLM7‐2	GLM7‐3	GLM7‐4	
	Total	< 7.0	7.0–7.5	7.5–8.0	>8.0	*p* value
	*n=26289*	*n=4931*	*n=9496*	*n=7960*	*n=3902*	
**Age**:						0.000
20–44	10557 (40.2%)	3195 (64.8%)	5205 (54.8%)	1653 (20.8%)	504 (12.9%)	
45–64	9137 (34.8%)	1072 (21.7%)	3041 (32.0%)	3524 (44.3%)	1500 (38.4%)	
>=65	6595 (25%)	664 (13.47%)	1250 (13.17%)	2783 (35.01%)	1898 (48.6%)	
Male:	12686 (48.3%)	2071 (42.0%)	4504 (47.4%)	3950 (49.6%)	2161 (55.4%)	<0.001
**Education**:						<0.001
<High school	2341 (8.9%)	245 (5.0%)	689 (7.3%)	906 (11.4%)	501 (12.9%)	
>=High school	23908 (91.1%)	4680 (95.0%)	8798 (92.6%)	7024 (88.6%)	3388 (87.1%)	
**Race**:						<0.001
Mexican American	3554 (13.5%)	479 (9.71%)	1309 (13.8%)	1152 (14.5%)	614 (15.7%)	
Other Hispanic	2733 (10.4%)	416 (8.44%)	1009 (10.6%)	894 (11.2%)	414 (10.6%)	
Non‐Hispanic White	9487 (36.1%)	1740 (35.3%)	3110 (32.8%)	2901 (36.4%)	1736 (44.5%)	
Non‐Hispanic Black	6132 (23.3%)	1329 (27.0%)	2244 (23.6%)	1815 (22.8%)	744 (19.1%)	
Other Race	4383 (16.7%)	967 (19.6%)	1824 (19.2%)	1198 (15.1%)	394 (10.1%)	
**Married**:	15354 (58.5%)	2553 (51.8%)	5811 (61.3%)	4723 (59.4%)	2267 (58.2%)	<0.001
**Alcohol**:	4963 (18.9%)	1083 (22.0%)	2572 (27.1%)	1076 (13.5%)	232 (5.95%)	<0.001
**Smoke**:						<0.001
Never	15168 (57.7%)	3097 (62.9%)	5878 (61.9%)	4286 (53.9%)	1907 (48.9%)	
Former	6177 (23.5%)	838 (17.0%)	1690 (17.8%)	2225 (28.0%)	1424 (36.5%)	
Current	4926 (18.8%)	992 (20.1%)	1921 (20.2%)	1443 (18.1%)	570 (14.6%)	
**Sport_level**:						<0.001
Inactive	14867 (56.6%)	2620 (53.1%)	5176 (54.5%)	4654 (58.5%)	2417 (61.9%)	
Moderate	5586 (21.2%)	1169 (23.7%)	1939 (20.4%)	1707 (21.4%)	771 (19.8%)	
Vigorous	1118 (4.25%)	206 (4.18%)	479 (5.04%)	303 (3.81%)	130 (3.33%)	
Moderate and vigorous	4718 (17.9%)	936 (19.0%)	1902 (20.0%)	1296 (16.3%)	584 (15.0%)	
**CVD (%)**:	3097 (11.8%)	308 (6.25%)	345 (3.63%)	1293 (16.2%)	1151 (29.5%)	0.000
**Diabetes**:	5136 (19.5%)	286 (5.80%)	469 (4.94%)	2135 (26.8%)	2246 (57.6%)	0.000
**Liver disease**:	1249 (4.75%)	116 (2.35%)	175 (1.84%)	588 (7.39%)	370 (9.48%)	<0.001
**Cancer**:	2690 (10.2%)	269 (5.46%)	363 (3.82%)	1213 (15.2%)	845 (21.7%)	<0.001
**Other comorbid**:	4582 (17.4%)	487 (9.88%)	612 (6.44%)	2087 (26.2%)	1396 (35.8%)	0.000

### 2.2 Univariate Analysis Revealed the Predictive Performance of 49 Indicators Across Various Diseases

**Figure 2:**
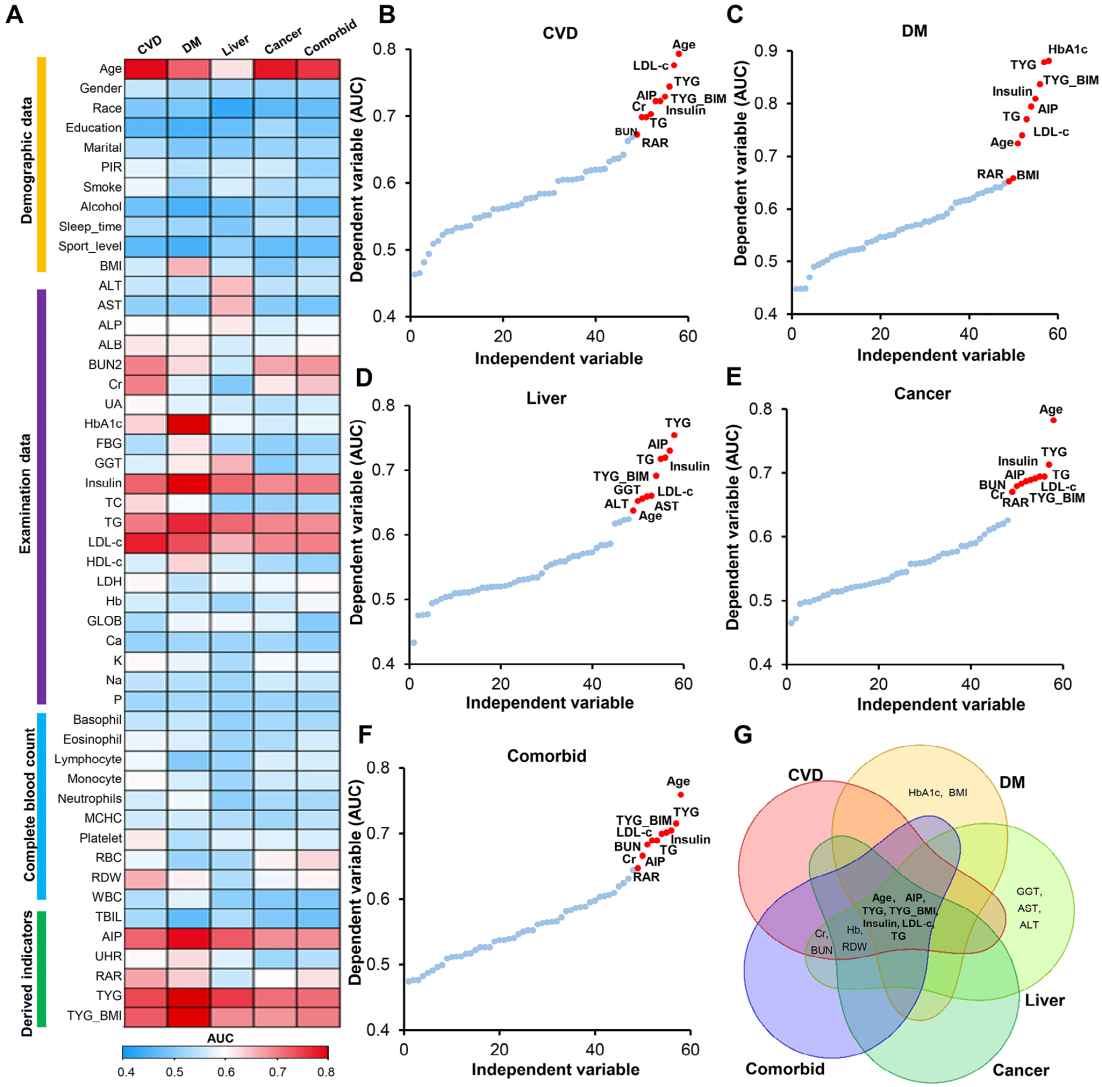
Univariate regression analysis of 49 factors on five major disease categories. A) Heatmap displaying the area under the curve (AUC) values derived from receiver operating characteristic (ROC) analysis for the association between 49 factors and various diseases. B) Univariate analysis of 49 factors for CVD and identification of the top 10 associated factors. C) Univariate analysis of 49 factors for DM and identification of the top 10 associated factors. D) Univariate analysis of 49 factors for liver diseases and identification of the top 10 associated factors. E) Univariate analysis of 49 factors for cancer and identification of the top 10 associated factors. F) Univariate analysis of 49 factors for comorbid conditions and identification of the top 10 associated factors. G) Venn diagram illustrating the overlap of significant factors identified through univariate analysis across different disease categories.

In this study, we constructed receiver operating characteristic (ROC) analysis to depict the independent impact of 49 individual variables on different diseases, utilizing the AUC as an enhanced quantitative metric (**Table** **2**; Tables S2–S6, Supporting Information). Subsequently, we organized the results into four major categories: Demographic data, Examination data, Complete blood count, and Derived indicators. We observed that factors such as age, insulin, triglycerides (TG), low‐density lipoprotein cholesterol (LDL‐c), atherogenic index of plasma (AIP), triglyceride‐glucose index (TYG), and triglyceride‐glucose‐body mass index (TYG_BMI) demonstrated excellent classification performance across multiple diseases (**Figure** **2**A). Specifically, the most informative 10 factors with the best classification effects for CVD were age, LDL‐c, TYG, TYG_BMI, AIP, Insulin, creatinine (Cr), blood urea nitrogen (BUN), TG, and red cell distribution width to albumin ratio (RAR) (Figure 2B). For DM, the most informative 10 factors were HbA1c, TYG, TYG_BMI, insulin, AIP, TG, LDL‐c, age, body mass index (BMI), and RAR (Figure 2C). In the case of liver disease, the top 10 factors were TYG, AIP, TG, insulin, TYG_BMI, gamma‐glutamyl transferase (GGT), LDL‐c, alanine aminotransferase (ALT), aspartate aminotransferase (AST), and age (Figure 2D). For cancer, the top 10 factors were age, TYG, TG, insulin, LDL‐c, AIP, BUN, Cr, TYG_BMI, and RAR (Figure 2E). Regarding other comorbidities, the most informative 10 factors were age, TYG, TYG_BMI, LDL‐c, TG, insulin, BUN, AIP, and RAR (Figure 2F). The majority of these results align with well‐known indicators and previous studies,^[^ ^17^, ^18^ ^]^ underscoring the robust stability of these factors in disease diagnosis. In particular, through a Venn diagram analysis of the top most informative influencing factors for the aforementioned five categories of diseases, we discovered that seven factors—age, AIP, TYG, TYG_BMI, LDL‐c, TG, and insulin—exhibited excellent classification effects across all diseases (Figure 2G). This not only further confirms the robustness of these routine detection indicators in predicting disease classification but also hints at their significant potential in disease diagnosis and prognosis.

**Table 2:** Univariate regression analysis of routine health indicators on five major disease categories.

ID	CVD‐AUC [95% CI]	DM‐ AUC [95% CI]	Liver‐ AUC [95% CI]	Cancer‐ AUC [95% CI]	Comorbid‐ AUC [95% CI]
Age	0.793 (0.786–0.801)	0.724 (0.718–0.731)	0.623 (0.606–0.64)	0.782 (0.774–0.791)	0.759 (0.752–0.767)
Gender	0.551 (0.541–0.56)	0.523 (0.515–0.531)	0.521 (0.507–0.535)	0.51 (0.5–0.52)	0.511 (0.503–0.519)
Race	0.494 (0.485–0.503)	0.49 (0.481–0.498)	0.433 (0.416–0.449)	0.465 (0.456–0.474)	0.474 (0.466–0.482)
Education	0.463 (0.455–0.471)	0.448 (0.441–0.455)	0.475 (0.462–0.487)	0.524 (0.517–0.532)	0.491 (0.485–0.498)
Marital	0.535 (0.526–0.545)	0.494 (0.487–0.502)	0.501 (0.487–0.515)	0.514 (0.504–0.524)	0.522 (0.515–0.53)
PIR	0.577 (0.567–0.587)	0.547 (0.539–0.556)	0.559 (0.543–0.575)	0.559 (0.548–0.57)	0.512 (0.503–0.521)
Smoke	0.584 (0.574–0.593)	0.513 (0.505–0.52)	0.567 (0.552–0.582)	0.538 (0.528–0.548)	0.54 (0.532–0.548)
Alcohol	0.481 (0.474–0.488)	0.449 (0.443–0.455)	0.477 (0.466–0.488)	0.514 (0.504–0.523)	0.476 (0.47–0.483)
Sleep_time	0.533 (0.522–0.545)	0.518 (0.509–0.527)	0.494 (0.477–0.511)	0.544 (0.533–0.556)	0.535 (0.525–0.544)
Sport_level	0.465 (0.455–0.474)	0.448 (0.44–0.455)	0.51 (0.495–0.525)	0.472 (0.463–0.482)	0.476 (0.468–0.484)
BMI	0.561 (0.55–0.571)	0.658 (0.65–0.666)	0.55 (0.534–0.566)	0.498 (0.487–0.509)	0.537 (0.528–0.546)
ALT	0.552 (0.542–0.562)	0.542 (0.533–0.551)	0.652 (0.634–0.669)	0.545 (0.534–0.556)	0.552 (0.543–0.561)
AST	0.509 (0.498–0.52)	0.503 (0.493–0.512)	0.656 (0.639–0.674)	0.501 (0.49–0.512)	0.487 (0.478–0.496)
ALP	0.603 (0.592–0.614)	0.601 (0.592–0.61)	0.617 (0.604–0.63)	0.565 (0.554–0.577)	0.587 (0.578–0.596)
ALB	0.62 (0.61–0.631)	0.616 (0.607–0.624)	0.564 (0.547–0.58)	0.577 (0.566–0.588)	0.606 (0.597–0.615)
BUN2	0.698 (0.687–0.709)	0.63 (0.621–0.639)	0.557 (0.54–0.574)	0.67 (0.659–0.682)	0.683 (0.674–0.692)
Cr	0.698 (0.687–0.708)	0.57 (0.56–0.58)	0.497 (0.48–0.514)	0.618 (0.607–0.63)	0.647 (0.637–0.657)
UA	0.606 (0.595–0.616)	0.576 (0.567–0.585)	0.559 (0.542–0.575)	0.542 (0.531–0.553)	0.564 (0.555–0.574)
HbA1c	0.636 (0.625–0.647)	0.881 (0.873–0.888)	0.584 (0.567–0.6)	0.562 (0.55–0.574)	0.583 (0.574–0.593)
FBG	0.533 (0.523–0.544)	0.621 (0.611–0.63)	0.53 (0.514–0.546)	0.508 (0.497–0.519)	0.517 (0.508–0.525)
GGT	0.569 (0.558–0.58)	0.617 (0.608–0.626)	0.659 (0.642–0.676)	0.503 (0.492–0.514)	0.537 (0.528–0.546)
Insulin	0.722 (0.709–0.734)	0.809 (0.8–0.818)	0.719 (0.699–0.738)	0.691 (0.678–0.704)	0.704 (0.694–0.714)
TC	0.632 (0.621–0.643)	0.591 (0.582–0.6)	0.511 (0.494–0.528)	0.515 (0.503–0.527)	0.526 (0.517–0.536)
TG	0.703 (0.691–0.715)	0.77 (0.761–0.779)	0.717 (0.699–0.735)	0.694 (0.682–0.707)	0.689 (0.679–0.699)
LDL–c	0.776 (0.766–0.786)	0.739 (0.73–0.748)	0.66 (0.643–0.677)	0.694 (0.683–0.705)	0.699 (0.69–0.708)
HDL–c	0.567 (0.556–0.578)	0.635 (0.627–0.644)	0.567 (0.551–0.584)	0.529 (0.517–0.541)	0.512 (0.502–0.521)
LDH	0.607 (0.597–0.618)	0.547 (0.538–0.556)	0.584 (0.568–0.601)	0.586 (0.574–0.597)	0.604 (0.595–0.613)
Hb	0.564 (0.553–0.575)	0.55 (0.541–0.559)	0.52 (0.503–0.537)	0.558 (0.547–0.569)	0.588 (0.579–0.597)
GLOB	0.528 (0.517–0.539)	0.584 (0.575–0.593)	0.586 (0.569–0.603)	0.574 (0.563–0.586)	0.499 (0.49–0.509)
Ca	0.513 (0.502–0.524)	0.521 (0.512–0.53)	0.518 (0.501–0.535)	0.522 (0.51–0.533)	0.509 (0.5–0.518)
K	0.605 (0.594–0.616)	0.58 (0.571–0.589)	0.531 (0.514–0.549)	0.59 (0.578–0.602)	0.586 (0.577–0.596)
Na	0.547 (0.536–0.559)	0.538 (0.529–0.547)	0.52 (0.503–0.537)	0.56 (0.549–0.572)	0.552 (0.543–0.561)
P	0.522 (0.511–0.533)	0.525 (0.516–0.534)	0.515 (0.498–0.531)	0.518 (0.507–0.529)	0.516 (0.507–0.525)
Basophil	0.548 (0.538–0.558)	0.551 (0.543–0.559)	0.51 (0.495–0.525)	0.526 (0.516–0.536)	0.526 (0.518–0.535)
Eosinophil	0.584 (0.573–0.594)	0.57 (0.561–0.579)	0.519 (0.503–0.535)	0.533 (0.522–0.544)	0.563 (0.554–0.572)
Lymphocyte	0.585 (0.574–0.596)	0.498 (0.489–0.507)	0.513 (0.496–0.53)	0.569 (0.557–0.581)	0.566 (0.556–0.576)
Monocyte	0.605 (0.595–0.616)	0.566 (0.557–0.575)	0.518 (0.502–0.535)	0.558 (0.547–0.57)	0.561 (0.552–0.57)
Neutrophils	0.562 (0.552–0.573)	0.585 (0.576–0.594)	0.505 (0.488–0.522)	0.528 (0.517–0.539)	0.526 (0.517–0.536)
MCHC	0.561 (0.55–0.572)	0.562 (0.553–0.572)	0.534 (0.518–0.551)	0.523 (0.512–0.534)	0.546 (0.537–0.555)
Platelet	0.617 (0.606–0.628)	0.536 (0.527–0.545)	0.571 (0.553–0.589)	0.574 (0.562–0.585)	0.565 (0.556–0.575)
RBC	0.584 (0.573–0.595)	0.516 (0.507–0.525)	0.526 (0.509–0.543)	0.597 (0.586–0.608)	0.624 (0.615–0.633)
RDW	0.663 (0.653–0.673)	0.614 (0.606–0.623)	0.534 (0.517–0.551)	0.586 (0.575–0.597)	0.609 (0.6–0.618)
WBC	0.536 (0.525–0.547)	0.576 (0.567–0.585)	0.504 (0.487–0.521)	0.498 (0.487–0.51)	0.496 (0.487–0.505)
TBIL	0.527 (0.516–0.538)	0.47 (0.462–0.479)	0.532 (0.515–0.549)	0.495 (0.484–0.506)	0.482 (0.473–0.491)
AIP	0.722 (0.711–0.733)	0.794 (0.786–0.802)	0.73 (0.714–0.746)	0.689 (0.677–0.7)	0.689 (0.679–0.698)
UHR	0.605 (0.595–0.616)	0.628 (0.619–0.636)	0.572 (0.556–0.589)	0.52 (0.509–0.532)	0.539 (0.53–0.549)
RAR	0.672 (0.662–0.682)	0.641 (0.633–0.65)	0.554 (0.538–0.571)	0.611 (0.599–0.622)	0.631 (0.623–0.64)
TYG	0.744 (0.733–0.755)	0.878 (0.872–0.885)	0.754 (0.738–0.77)	0.713 (0.701–0.724)	0.715 (0.705–0.724)
TYG_BMI	0.729 (0.718–0.74)	0.836 (0.829–0.844)	0.691 (0.673–0.708)	0.683 (0.671–0.695)	0.701 (0.692–0.711)
GLM7	0.933 (0.895–0.971)	0.966 (0.948–0.984)	0.894 (0.855–0.933)	0.887(0.878–0.895)	0.867(0.861–0.876)

### 2.3 Overall Trends of 7 Routine Indicators and the Relationship Between GLM7 and Disease

**Figure 3:**
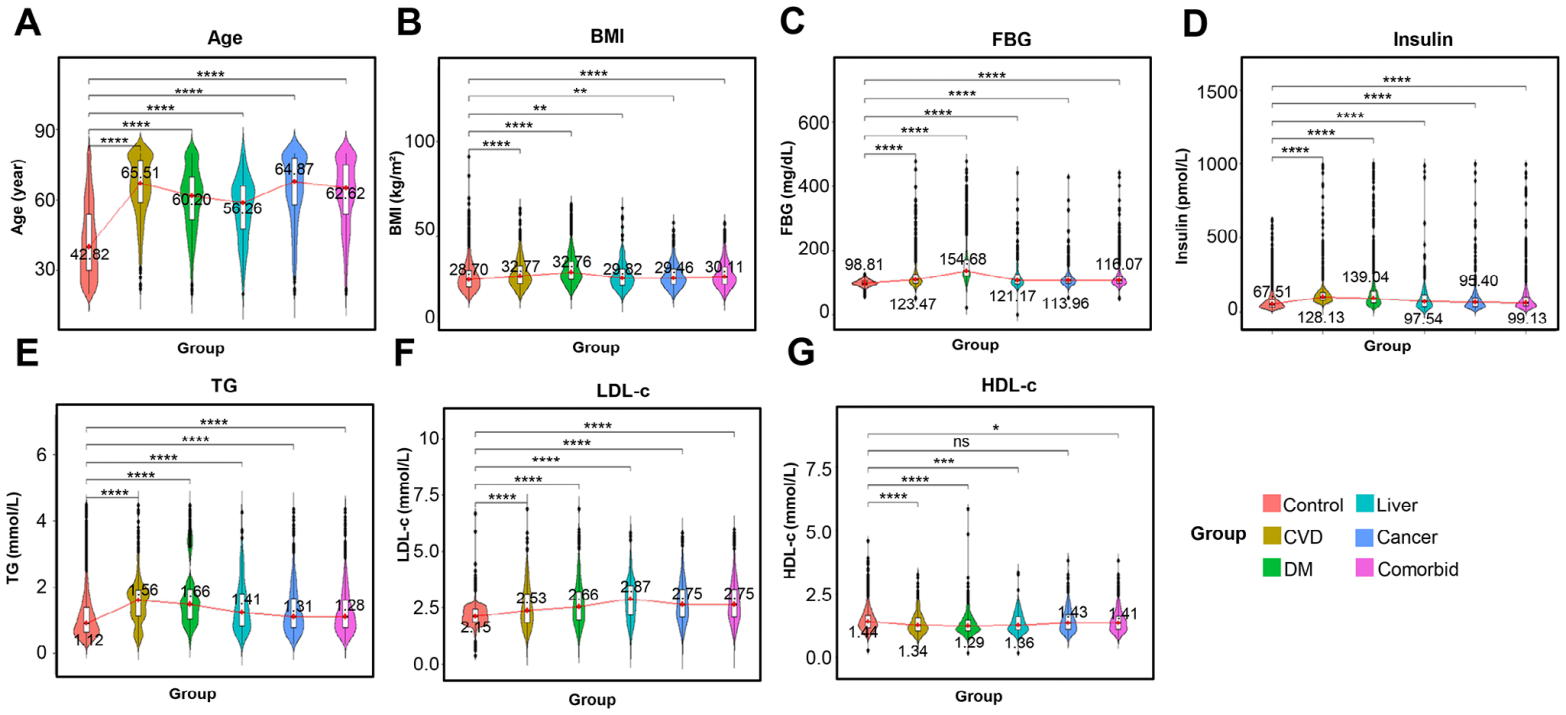
Relative trends of seven common factors across normal and disease populations. A) Mean values and trends of Age in the normal population and subgroups with various diseases. B) Mean values and trends of body mass index (BMI) in the normal population and disease subgroups. C) Mean values and trends of fasting blood glucose (FBG) in the normal population and disease subgroups. D) Mean values and trends of insulin levels in the normal population and disease subgroups. E) Mean values and trends of triglycerides (TG) in the normal population and disease subgroups. F) Mean values and trends of low‐density lipoprotein‐cholesterol (LDL‐c) in the normal population and disease subgroups. G) Mean values and temporal trends of high‐density lipoprotein‐cholesterol (HDL‐c) in the normal population and disease subgroups.

Given that several factors, including AIP, TYG, and TYG_BMI, are primarily derived from routine detection indicators such as TG, fasting blood glucose (FBG), BMI, and high‐density lipoprotein cholesterol (HDL‐c), we ultimately focused on exploring the impact of seven routine detection indicators—age, TG, FBG, BMI, HDL‐c, LDL‐c, and insulin—on various diseases to simplify the quantification of these indicators. Statistical analysis using the “survey” package revealed that six factors—age, TG, FBG, BMI, LDL‐c, and insulin—were significantly higher in several disease groups compared to the healthy population (**Figure** **3**A–F). In contrast, HDL‐c levels were significantly lower in individuals with CVD, DM, liver disease, and various complications (Figure 3G). These findings are generally consistent with previous studies. The mean values of these indicators across different populations are presented in Table S7 (Supporting Information).

To more clearly demonstrate the influence of these seven factors on diseases, we introduced a new indicator, GLM7, with the following calculation formula:

(1) GLM7=log10Ageyear×BMIkg/m2×FBGmg/dL×Insulinpmol/L×TGmmol/L×LDL−cmmol/LHDL−cmmol/L

**Figure 4:**
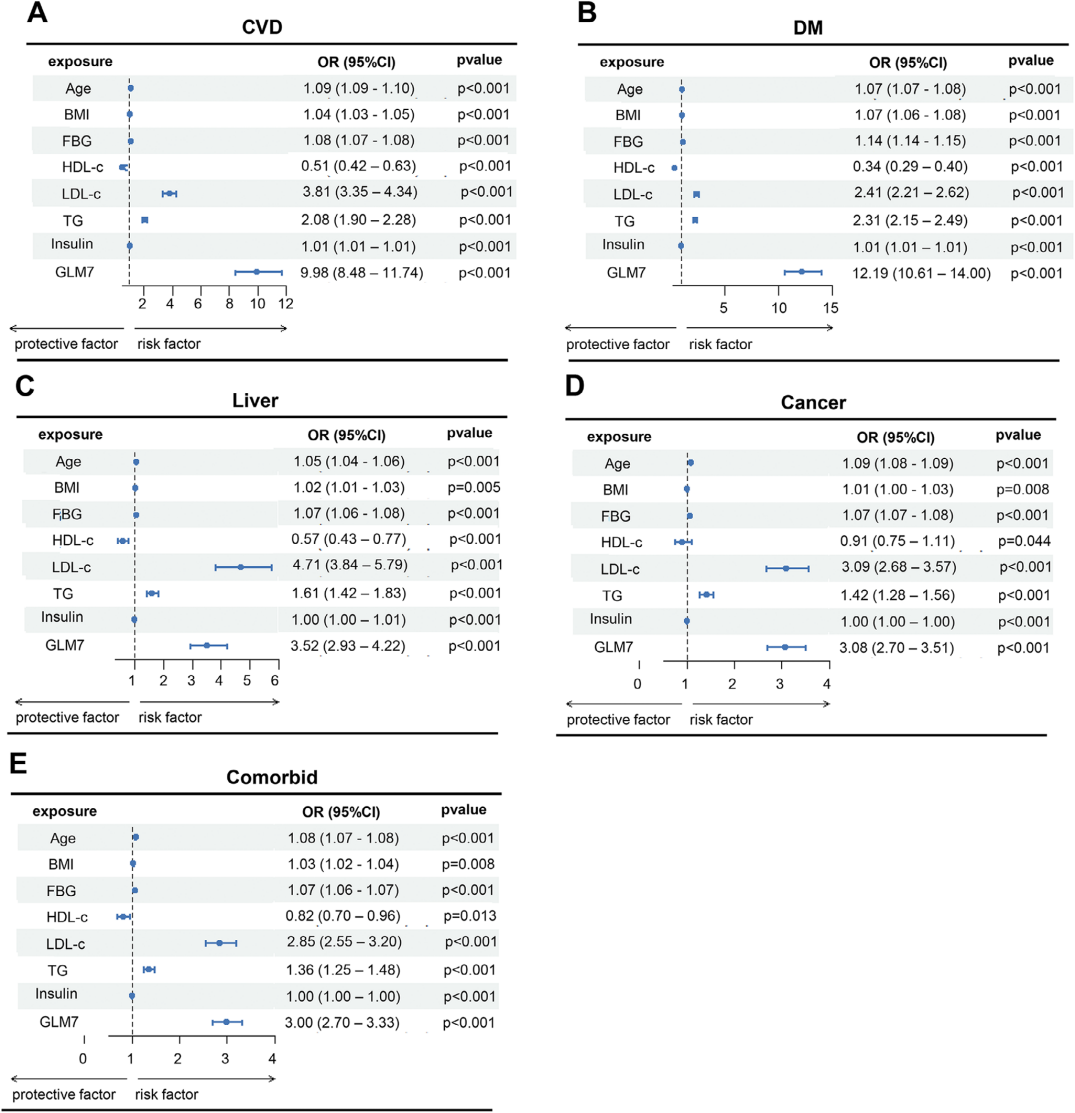
Forest plots depicting the associations between conventional factors, their integrated indices GLM7, and the incidence of various diseases. A) Forest plot illustrating the relationships between individual factors and the incidence of CVD. B) Forest plot illustrating the relationships between individual factors and the incidence of DM. C) Forest plot illustrating the relationships between individual factors and the incidence of liver diseases. D) Forest plot illustrating the relationships between individual factors and the incidence of cancer. E) Forest plot illustrating the relationships between individual factors and the incidence of comorbid conditions.

Univariate logistic regression analysis results indicated that the odds ratio (OR) of GLM7 for CVD was 9.98 (95% CI: 8.48–11.74) (**Figure** **4**A); for DM, the OR was 12.19 (95% CI: 10.61–14.00) (Figure 4B); for liver disease, the OR was 3.52 (95% CI: 2.93–4.22) (Figure 4C); for cancer, the OR was 3.08 (95% CI: 2.70–3.51) (Figure 4D); and for other complications, the OR was 3.00 (95% CI: 2.70–3.33) (Figure 4E). These results not only demonstrate the robustness of GLM7, a new indicator derived from the seven routine detection indicators (age, TG, FBG, BMI, HDL‐c, LDL‐c, and insulin), in disease diagnosis but also suggest its significant potential as a novel diagnostic and predictive tool for diseases in the future.

### 2.4 Contribution of Specific GLM7 Domains to Different Disease Events

**Figure 5:**
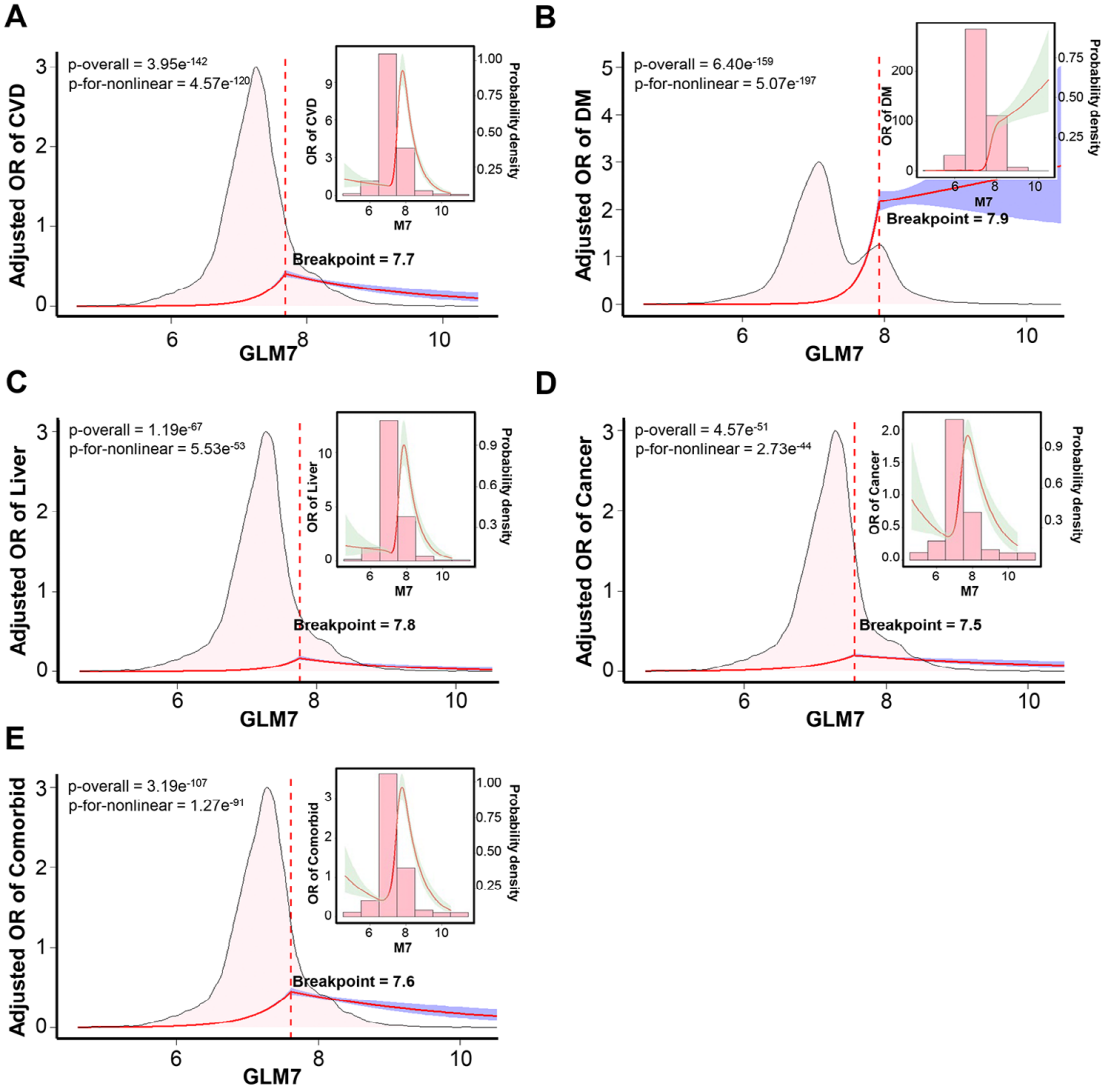
Restricted cubic splines (RCS) analysis of the association between the GLM7 index and disease incidence across different disease categories. A) RCS analysis illustrating the nonlinear relationship between the GLM7 index and the incidence of CVD. B) RCS analysis illustrating the nonlinear relationship between the GLM7 index and the incidence of DM. C) RCS analysis illustrating the nonlinear relationship between the GLM7 index and the incidence of liver diseases. D) RCS analysis illustrating the nonlinear relationship between the GLM7 index and the incidence of cancer. E) RCS analysis illustrating the nonlinear relationship between the GLM7 index and the incidence of comorbid conditions.

To further explore the relationship between GLM7 and various diseases, we employed RCS analysis to illustrate the associations between disease incidence and specific domain GLM7 scores across different disease populations. The analysis revealed a significant nonlinear relationship between GLM7 and CVD, with a risk threshold of 7.7, indicating an increased odds ratio for CVD risk beyond this value (**Figure** **5**A). Similarly, GLM7 demonstrated a notable nonlinear relationship with DM, having a risk threshold of 7.9 (Figure 5B). GLM7 also exhibited a clear nonlinear relationship with liver disease, with a risk threshold of 7.8 (Figure 5C), as well as with cancer, with a risk threshold of 7.5 (Figure 5D). Furthermore, GLM7 showed a significant nonlinear relationship with other complications, with a risk threshold of 7.6 (Figure 5E). In summary, GLM7 exhibits substantial nonlinear relationships with most diseases, and the odds ratio for developing any of the aforementioned diseases rises when GLM7 exceeds 7.5. These findings further validate the robustness of GLM7 in disease diagnosis and prediction, providing us with accurate reference intervals.

### 2.5 Subgroup Associations Between GLM7 Index and Disease Outcomes Across GLM7 Thresholds

**Figure 6:**
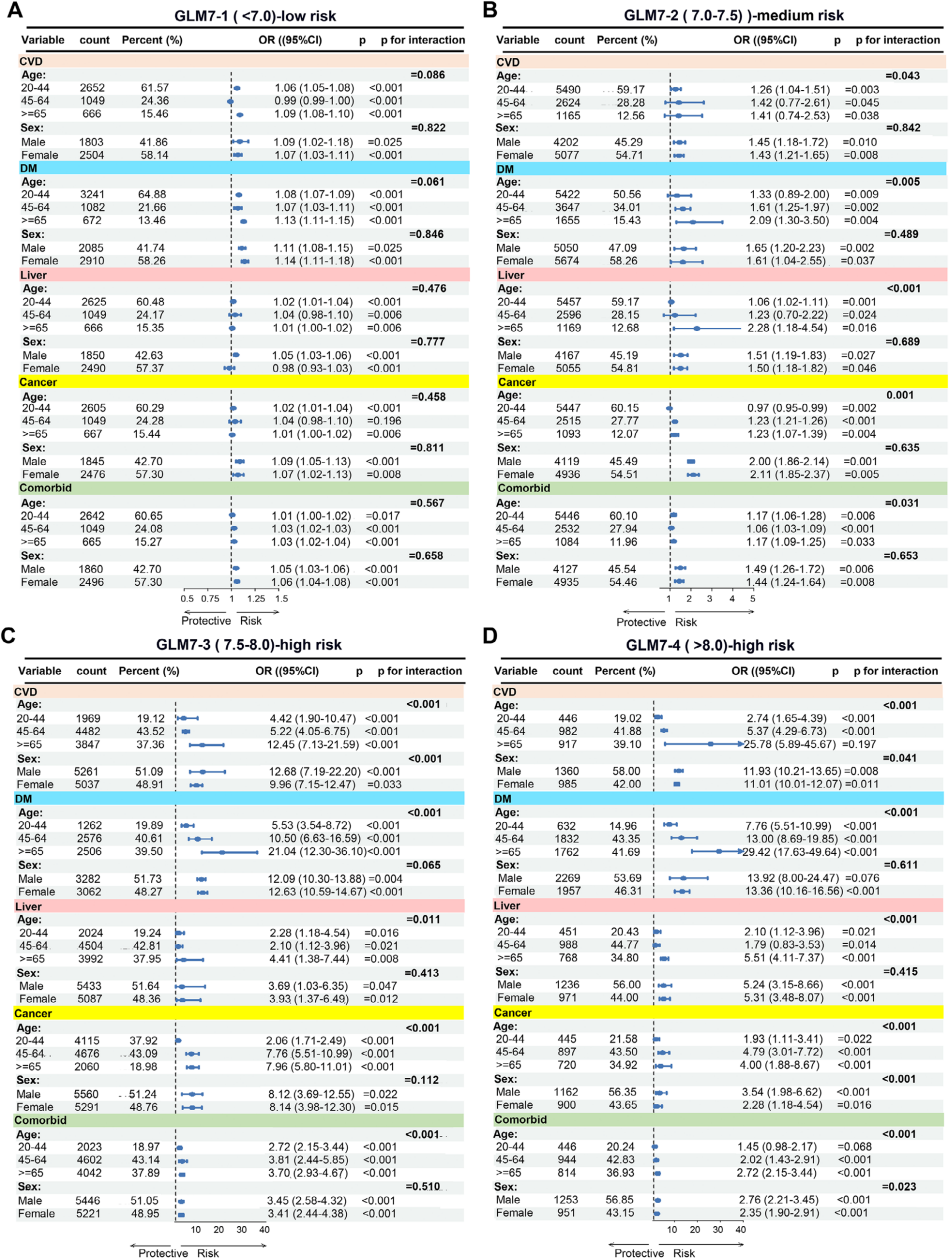
Subgroup analyses of the association between the GLM7 index and disease under different thresholds of GLM7. A) Risk assessment of the GLM7 index for five types of diseases across different ages and genders when GLM7 < 7.0. B) Risk assessment of the GLM7 index for five types of diseases across different ages and genders when 7.0 ≤ GLM7 < 7.5. C) Risk assessment of the GLM7 index for five types of diseases across different ages and genders when 7.5 ≤ GLM7 < 8.0. D) Risk assessment of the GLM7 index for five types of diseases across different ages and genders when GLM7 ≥ 8.0.

In order to delve deeper into how GLM7 influences diverse disease subgroups across varying threshold ranges, we conducted interaction tests based on grouping factors such as age, sex, CVD, DM, liver disease, cancer, and various comorbidities. The results are presented in **Figure** **6**. The analysis revealed that GLM7 serves as a risk factor for various diseases across different threshold ranges, with the risk of disease occurrence increasing as the value of GLM7 rises (Figure 6A–D). Specifically, when the value of GLM7 is less than 7.0, GLM7 has an impact on various diseases, but the overall OR is not notably high, except for OR values exceeding 1.1 in male and female DM subgroups and individuals aged over 65 years, while all other OR values remain below 1.1, with no significant differences observed among different age and sex subgroups (Figure 6A). When the value of GLM7 ranges from 7.0 to 7.5, the risk ratio of GLM7 for various diseases begins to increase, with a notably elevated incidence risk of CVD, DM, liver disease, cancer, and comorbidities observed in individuals aged over 65 years, while no significant changes are noted in male and female subgroups (Figure 6B). When the value of GLM7 ranges from 7.5 to 8.0, the impact of GLM7 on various diseases sharply increases, with a clear age‐related rise in incidence risk across all age groups (Figure 6C). This suggests that a GLM7 value of 7.5 may represent a critical threshold for clinical diagnosis, aligning with the findings of the preceding RCS analysis (Figure 5). When the value of GLM7 exceeds 8.0, the impact of GLM7 on CVD and DM continues to rise, with evident age stratification. However, the effects on liver disease, cancer, and other comorbidities tend to stabilize (Figure 6D). Overall, when the GLM7 value is less than 7.0, it has a relatively minor impact on the risk of various diseases; when the GLM7 value falls within the range of 7.0‐7.5, the risk of developing various diseases increases; when the GLM7 threshold exceeds 7.5, the incidence risk of various diseases sharply rises. Additionally, age‐stratified results also reveal that when GLM7 surpasses the medium‐to‐high risk threshold, older individuals exhibit a higher incidence risk within the same GLM7 threshold range. These findings offer guidance for the more refined application of the GLM7 indicator in clinical diagnosis.

### 2.6 Performance of a 7‐Factor Machine Learning Model in the Diagnostic Prediction of Various Diseases

**Figure 7:**
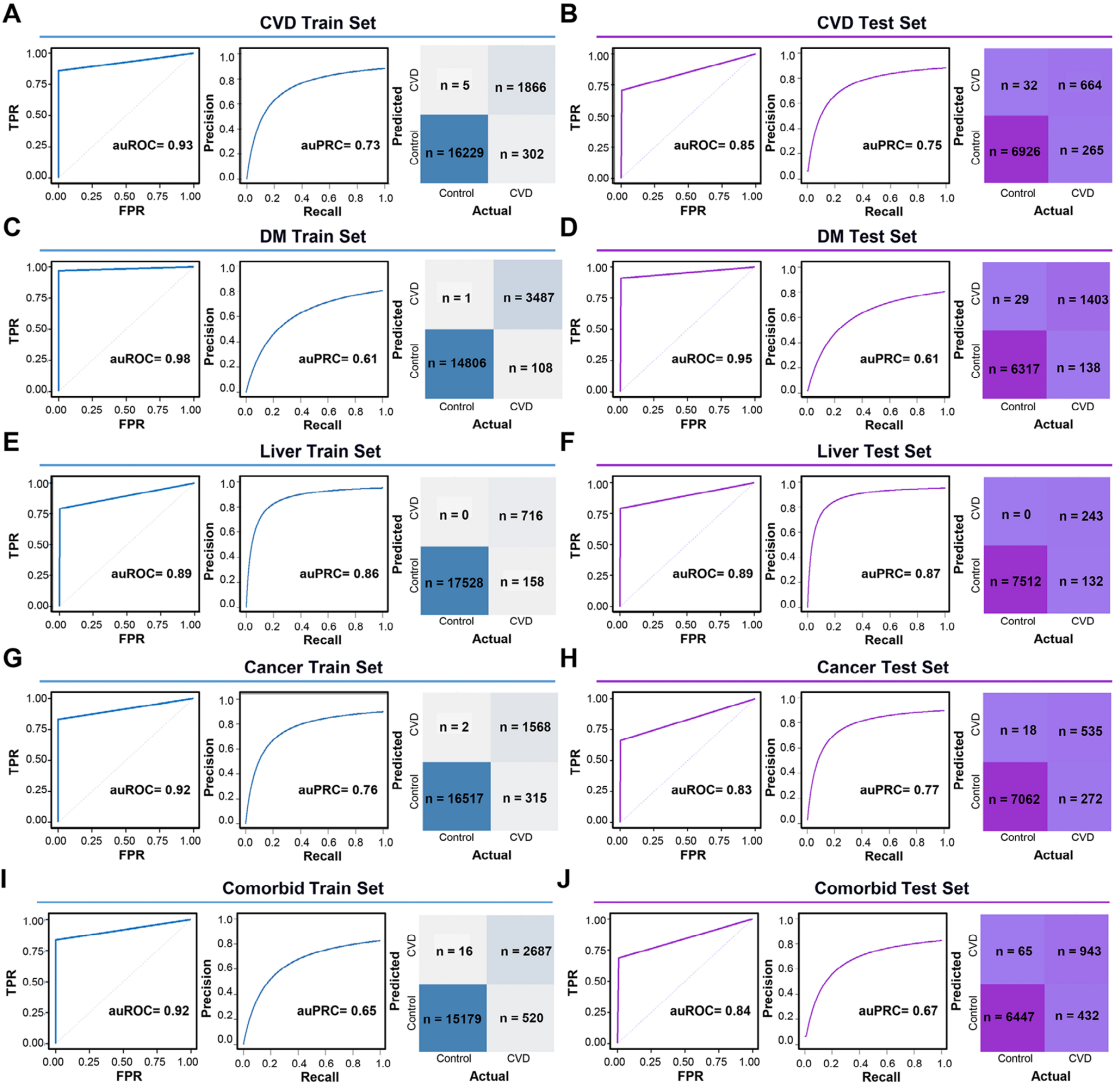
Predictive performance of machine learning models in training cohorts and diagnostic performance of the final model in validation cohorts. A) Performance evaluation of the machine learning model in the training cohort for CVD using receiver operating characteristic (ROC) curves, precision‐recall curves (PRC), and confusion matrices. B) Performance evaluation of the final machine learning model in the validation cohort for CVD using ROC curves, PRC, and confusion matrices. C) Performance evaluation of the machine learning model in the training cohort for DM using ROC curves, PRC, and confusion matrices. D) Performance evaluation of the final machine learning model in the validation cohort for DM using ROC curves, PRC, and confusion matrices. E) Performance evaluation of the machine learning model in the training cohort for liver diseases using ROC curves, PRC, and confusion matrices. F) Performance evaluation of the final machine learning model in the validation cohort for liver diseases using ROC curves, PRC, and confusion matrices. G) Performance evaluation of the machine learning model in the training cohort for cancer using ROC curves, PRC, and confusion matrices. H) Performance evaluation of the final machine learning model in the validation cohort for cancer using ROC curves, PRC, and confusion matrices. I) Performance evaluation of the machine learning model in the training cohort for comorbid conditions using ROC curves, PRC, and confusion matrices. J) Performance evaluation of the final machine learning model in the validation cohort for comorbid conditions using ROC curves, PRC, and confusion matrices.

Given the good stability of the aforementioned routine detection indicators in disease classification, we developed an XGBoost machine learning model based on seven factors for rapid disease screening and diagnosis, the shapley additive explanations (SHAP) values for explaining the importance of pivotal features in determining the weight were shown in Figure S1 (Supporting Information). The data from NHANES were divided into training and test sets at a 0.7 ratio, while data from CHARLS were utilized as an external validation cohort. The area under the ROC curve (auROC) was used to quantify the overall performance of the model. The area under the precision‐recall curve (auPRC) illustrated the trade‐off between precision and recall at different thresholds. The confusion matrix provided a detailed classification of the model's predictive results. The results showed that for CVD prediction, the model achieved an auROC of 0.93 and an auPRC of 0.73 in the training set. The confusion matrix also indicated that the vast majority of true positive and true negative results were accurately predicted (**Figure** **7**A). In the test set, the auROC was 0.85, and the auPRC was 0.75 (Figure 7B). These results demonstrated the good performance of the model in predicting CVD. Applying the same model to DM, the training set achieved an auROC of 0.98 and an auPRC of 0.61 (Figure 7C), while the test set achieved an auROC of 0.95 and an auPRC of 0.61 (Figure 7D). This indicated an even better predictive effect of the model for DM. Similarly, for liver disease, the training set achieved an auROC of 0.89 and an auPRC of 0.86 (Figure 7E), while the test set achieved an auROC of 0.89 and an auPRC of 0.87 (Figure 7F), showing superior predictive performance. In cancer prediction, the model achieved an auROC of 0.92 and an auPRC of 0.76 in the training set (Figure 7G), and an auROC of 0.83 and an auPRC of 0.77 in the test set (Figure 7H), maintaining good predictive performance. For other complications, the model achieved an auROC of 0.92 and an auPRC of 0.65 in the training set (Figure 7I), and an auROC of 0.84 and an auPRC of 0.67 in the test set (Figure 7J). These results indicated the good performance of our machine learning model based on seven routine factors in predicting diseases.

### 2.7 Validation of 7‐Factor Machine Learning Model in Cohort from CHARLS

**Figure 8:**
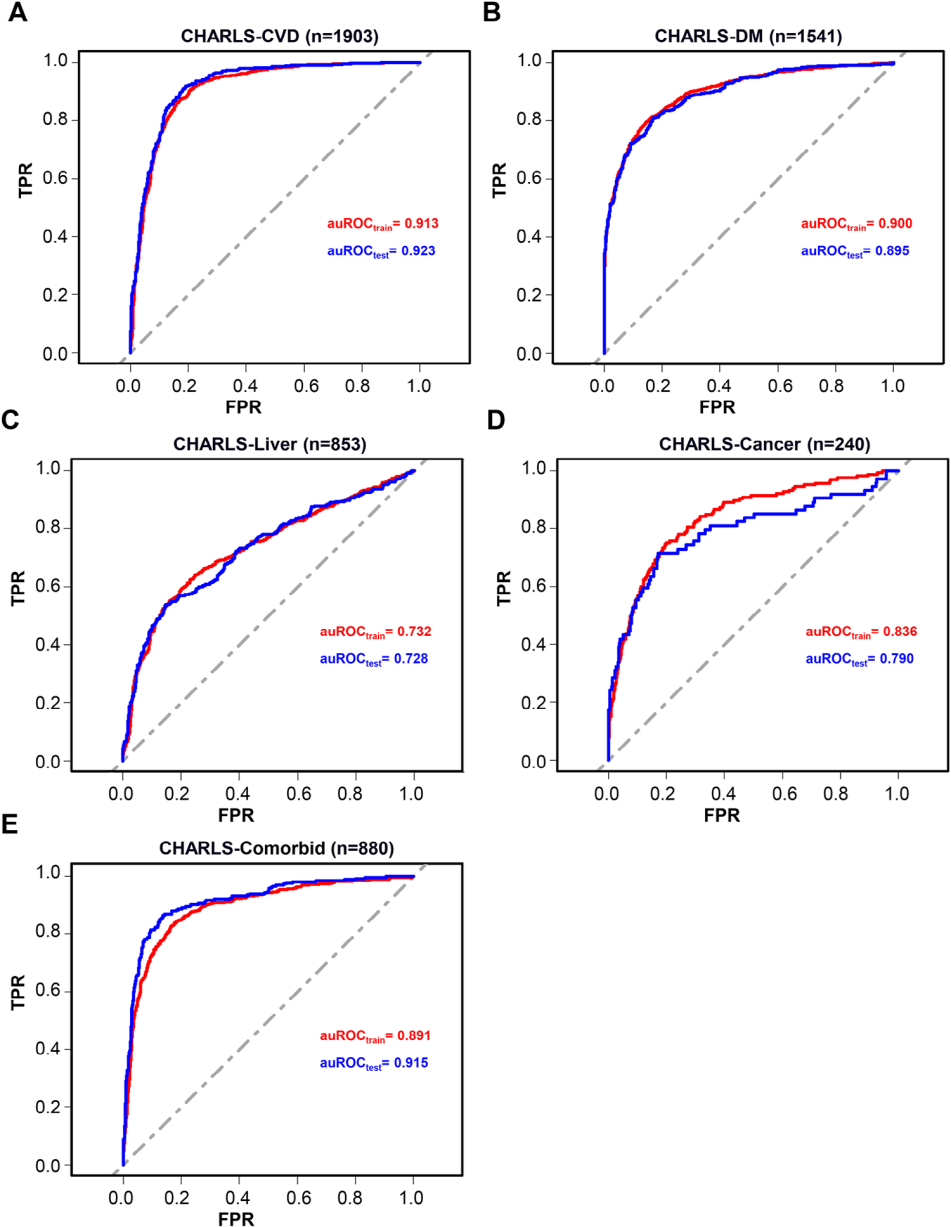
Performance of GLM7 model for diagnosis in external cohorts. A) ROC curve for GLM7 in discriminating CVD from patients in CHARLS. B) ROC curve for GLM7 in discriminating DM from patients in CHARLS. C) ROC curve for GLM7 in discriminating liver diseases from patients in CHARLS. D) ROC curve for GLM7 in discriminating cancer from patients in CHARLS. E) ROC curve for GLM7 in discriminating comorbid conditions from patients in CHARLS.

To further exclude batch effects, we further validated the reliability of the aforementioned model in the CHARLS database. The final results showed that for CVD prediction in the CHARLS database, the training set achieved an auROC of 0.913, and the validation set achieved an auROC of 0.923 (**Figure** **8**A). For DM prediction, the training set achieved an auROC of 0.900, and the validation set achieved an auROC of 0.895 (Figure 8B). For liver disease prediction, the training set achieved an auROC of 0.732, and the validation set achieved an auROC of 0.728 (Figure 8C). For cancer prediction, the training set achieved an auROC of 0.836, and the validation set achieved an auROC of 0.790 (Figure 8D). For other complications prediction, the training set achieved an auROC of 0.891, and the validation set achieved an auROC of 0.915 (Figure 8E). These external validation cohort results further consolidate the predictive performance of our machine learning model. The aforementioned findings not only suggest the promising application prospects of our machine learning model based on seven routine factors in disease prediction but also provide a new means for precise disease diagnosis.

## 3 Discussion

Harnessing routine health examination data efficiently to predict the future onset of multiple diseases is critical, as it enables early risk stratification, personalized interventions, and optimized resource allocation within healthcare systems.^[^ ^19^, ^20^ ^]^ Given the escalating global burden of chronic diseases, integrating longitudinal physical examination data into predictive models can identify high‐risk individuals prior to symptom manifestation, thereby facilitating timely lifestyle adjustments or therapeutic interventions.^[^ ^21^, ^22^ ^]^ The present study provides compelling evidence for the utility of routine health examination indicators in disease diagnosis and prediction, while also introducing a novel composite index, GLM7, which integrates multiple routine factors to enhance predictive accuracy. Specifically, we identified the top 10 diagnostic and predictive indicators for each of the five disease categories (CVD, DM, liver diseases, cancers, and comorbidities) (Figure 2), as well as their composite index, GLM7—a novel index derived from seven basic indicators (age, TG, FBG, BMI, HDL‐c, LDL‐c, and insulin). Additionally, we preliminarily delineated the risk threshold range for GLM7, indicating that the risk of developing various diseases significantly increases when the index exceeds 7.5. These studies not only highlight the enduring value of conventional detection parameters but also provide new approaches for disease diagnosis. These findings are consistent with a substantial body of literature indicating that routine measurements such as blood pressure, cholesterol levels, and fasting blood glucose concentrations are reliable markers of disease risk.^[^ ^23^, ^24^, ^25^ ^]^ For instance, the consistent associations observed between LDL‐c and cardiovascular outcomes reaffirm its role as cornerstones in cardiovascular risk assessment.^[^ ^26^, ^27^ ^]^

The robustness of the conventional indicators observed in this study holds significant implications for clinical practice. It supports the continued inclusion of these tests in routine health examinations, while also emphasizing the need to prioritize these informative markers over less relevant ones. This can help streamline physical examinations, reduce unnecessary tests, and alleviate the economic burden on patients. Moreover, the stability of these indicators across different disease categories suggests that they can serve as universal screening tools, facilitating early detection and intervention for a variety of diseases.

Additionally, the introduction of GLM7, a composite index based on seven routine glycolipid metabolism factors, represents a significant advancement in the field. By integrating multiple relevant parameters, GLM7 captures the complex interplay between various pathophysiological processes, providing a more comprehensive assessment of disease risk than individual indicators alone. This approach aligns with the growing trend in precision medicine toward using multidimensional biomarkers to enhance diagnostic and predictive accuracy. Restricted cubic spline analysis provided valuable insights into the nonlinear and linear relationships between GLM7 and various diseases, as well as the identification of risk thresholds. Moreover, when compared with several well‐established composite indices, namely TYG, TYG_BMI, and AIP, GLM7 demonstrates distinct advantages in clinical diagnosis and decision‐making (Table 2; Figure S2, Supporting Information). These findings are crucial for establishing clinical cut‐offs and interpreting the clinical significance of GLM7 values. For example, the observed nonlinear relationship between GLM7 and the risk of liver diseases suggests that the impact of glycolipid metabolism disorders on liver health may vary across different ranges of the index, highlighting the need for nuanced interpretation rather than a one‐size‐fits‐all approach. The forest plots illustrating the risk associations between GLM7 and diseases visually represent the strength and consistency of these relationships. This summary can assist clinicians in understanding the relative contributions of each component of GLM7 to disease risk, facilitating more informed decision‐making in clinical practice.

Finally, the excellent predictive capability of the XGBoost model, based on seven routine testing indicators, in both the NHANES discovery and CHARLS validation cohorts underscores the powerful potential of machine learning techniques in leveraging routine health examination data for disease prediction. Unlike traditional statistical methods, machine learning models can capture complex nonlinear relationships among multiple variables, making them particularly suitable for analyzing the high‐dimensional data generated by routine health examinations. The validation of the XGBoost model in the CHARLS cohort, which represents a distinct ethnic population, enhances the generalizability of the study findings. This suggests that the model can be applied across different clinical settings, potentially improving disease prediction and prevention on a population‐wide scale. Thus, the integration of machine learning with routine health examination data holds immense promise for transforming preventive medicine, enabling more accurate risk stratification and personalized intervention strategies.

The findings of the present study have important implications for precision disease management. By confirming the utility of conventional indicators and introducing a novel composite index, GLM7, the study provides a foundation for developing more targeted and efficient health screening protocols. The identification of the most informative indicators can help prioritize tests in routine examinations, focusing on those that provide the greatest diagnostic value while eliminating unnecessary ones. Moreover, the development of the XGBoost predictive model offers a practical tool for integrating routine health examination data into clinical decision‐making. This model could be incorporated into electronic health records, providing real‐time risk assessments for various diseases and guiding clinicians in implementing appropriate preventive measures. Such an approach has the potential to shift the focus of healthcare from reactive treatment to proactive prevention, ultimately improving population health and reducing healthcare costs.

However, we recognize that there are several limitations in this study. First, the reliance on observational data from databases such as NHANES and CHARLS precludes causal inference. While the study demonstrates associations between indicators and diseases, it cannot establish causality. Second, the focus on routine examination indicators may have overlooked other potentially important biomarkers that are not typically included in standard physical examinations. Third, the diseases included in the study (CVD, DM, liver diseases, cancers, comorbidities) represent a broad range, but the analysis of specific subtypes within these categories was limited. Future studies could delve deeper into specific disease subtypes to provide more targeted insights, and should include larger representation of traditionally under‐represented populations. In addition, the potential mechanistic explanation regarding how GLM7 exerts its predictive efficacy also warrants further exploration.

## 4 Conclusion

The present study represents a significant step forward in the quest for optimizing routine health examinations and improve disease diagnosis and prediction. By leveraging the power of large‐scale epidemiological databases, advanced statistical techniques, and machine learning algorithms, we have been able to gain a deeper understanding of the relationships between routine testing indicators and diseases. Our findings not only confirm the value of conventional indicators but also introduce a novel indicator, GLM7, that holds promise for precision medicine. As we continue to refine and validate these findings, we anticipate that the present study will impact clinical practice and public health policy.

## 5 Experimental Section

### Study Design and Population

A total of 26 289 individuals were included in this cross‐sectional investigation, all of whom were participants in the National Health and Nutrition Examination Survey (NHANES) conducted over the period of 2013–2023. Within this group, 3097 individuals had been diagnosed with CVD, 5136 with DM, 1249 with liver disease, 2690 with cancer, and 4582 presented with other comorbid conditions. For external validation, a separate cohort of 19 328 participants was drawn from The China Health and Retirement Longitudinal Study (CHARLS), a nationwide survey spanning the years 2011–2020. This validation cohort comprised 1903 participants with CVD, 1541 with DM, 853 with liver diseases, 240 with cancer, and 880 with other comorbidities. The exclusion criteria for participation in the study were defined as: 1) individuals below the age of 20; 2) participants with incomplete data for conditions including angina pectoris (AP), coronary artery disease (CAD), heart attack (HA), heart failure (HF) and stroke; 3) subjects lacking data for DM; 4) those missing data for all forms of liver disease; 5) individuals missing data for any type of cancer; 6) participants with missing data for comorbidities such as failing kidneys, chronic bronchitis, thyroid problems, and rheumatoid disorders. The NHANES protocol was subjected to review and subsequently obtained ethical clearance from the National Center for Health Statistics Institutional Review Board. Detailed policies and information can be obtained from the official website: https://www.cdc.gov/nchs/nhanes/index.html. The CHARLS study was sanctioned by the Institutional Review Board of Peking University (IRB00001052‐11015), and all participants provided written informed consent before being enrolled in the research.^[^ ^28^ ^]^

### Data Extraction

Demographic data: age, race, gender, marital, education, poverty income ratio (PIR), smoke, alcohol, sleep_time, sport_level and body mass index (BMI, kg m^−2^).

Examination data: alanine aminotransferase (ALT: U/L), aspartate aminotransferase (AST: U/L), alkaline phosphatase (ALP: U/L), albumin (ALB: g/L), blood urea nitrogen (BUN: mg/dL), creatinine (Cr: µmol/L), uric acid (UA: µmol/L), glycohemoglobin (HbA1c: %), FBG (mg/dL), gamma‐glutamyl transferase (GGT: U/L), Insulin (pmol/L), TG (mmol/L), HDL‐c (mmol/L), LDL‐c (mmol/L), total cholesterol (TC: mmol/L), lactate dehydrogenase (LDH: U/L), hemoglobin (Hb: g/dL), globulin (GLOB: g/L), calcium (Ca: mmol/L), sodium (Na: mmol/L), potassium (K: mmol/L), phosphorus (P: mmol/L).

Complete blood count data: basophil (1000 /µL), eosinophil (1000 /µL), lymphocyte (1000 /µL), monocyte (1000 /µL), neutrophils (1000 /µL), mean corpuscular hemoglobin concentration (MCHC: g/dL), platelet (1000 /µL), red blood cell (RBC: 10 00 000 /µL), red cell distribution width (RDW: %), white blood cell (WBC: 1000 /µL), total bilirubin (TBIL µmol/L).

Derived indicators: AIP was calculated as log_10_[TG (mg/dL) / HDL (mg/dL)];^[^ ^29^ ^]^ UHR was calculated as [UA (mg/dL) / HDL (mg/dL)];^[^ ^30^ ^]^ RAR was calculated as [RDW (%) / WBC (1000 /µL)];^[^ ^31^ ^]^ TyG was calculated as ln [TG (mg/dL) ×FBG (mg/dL)/2];^[^ ^32^ ^]^ TYG_BMI was calculated as ln [TG (mg/dL) × FBG (mg/dL)/2] × BMI (kg m^−2^).^[^ ^33^ ^]^

### Definition of Data

CVD was defined as the presence of any of the following conditions: heart failure, stroke, coronary artery disease, heart attack (myocardial infarction), and angina pectoris, or as having a history of CVD. DM was defined as having a history of diabetes or an HbA1c level > 6.5%.^[^ ^34^ ^]^ Liver disease was defined as being diagnosed with any liver condition by a physician, including but not limited to nonalcoholic fatty liver disease, hepatitis, cirrhosis, and liver cancer. Cancer was defined as being diagnosed with any neoplastic disease by a physician, encompassing all types of cancer. Comorbid conditions were defined as the presence of any of the following conditions: failing kidneys, chronic bronchitis, thyroid problems, and rheumatoid disorders.

### Definitions of the Exposure and Outcome Variables

A total of 49 indicators, encompassing Demographic data, Examination data, Complete blood count data, and Derived indicators, were utilized as exposure variables in this study. The definitions and selections of all indicators strictly adhere to the standardized identifiers from the NHANES database, as detailed at https://wwwn.cdc.gov/nchs/nhanes/tutorials/default.aspx. Additionally, the GLM7 index was defined as a novel exposure variable, which was calculated according to the following formula: log_10_ [Age (years) * BMI (kg/m^2^) * FBG (mg/dL) * Insulin (pmol/L) * TG (mmol/L) * LDL‐c (mmol/L) / HDL‐c (mmol/L)]. The outcome variables included the incidence of all diseases, such as CVD, DM, liver disease, cancer, and comorbid conditions.

### GLM7 Formula

To develop a more universally applicable and easily computable formula, several well‐established indices, encompassing the calculation formulas for TYG = [ln [TG (mg/dL) × FBG (mg/dL)/2]], AIP = [log_10_[TG (mg/dL) / HDL‐c (mg/dL)]], TYG_BMI = [ln [TG (mg/dL) × FBG (mg/dL)/2] × BMI (kg/m^2^)], and RAR = [RDW (%) / WBC (1000 /µL)] were scrutinized. It was observed that all these indices are based on fundamental and widely accessible computational formulas. Building on this understanding and incorporating insights from univariate regression analysis, the GLM7 formula was refined to: log_10_ [Age (years) × BMI (kg/m^2^) × FBG (mg/dL) × Insulin (pmol/L) × TG (mmol/L) × LDL‐c (mmol/L) / HDL‐c (mmol/L)]. This formula is essentially derived by dividing the product of six factors positively correlated with the disease (Age, BMI, FBG, Insulin, TG, and LDL‐c) by the factor (HDL‐c) negatively associated with the disease, followed by a base‐10 logarithmic transformation. Given that the values generated by the original formula were excessively large and unwieldy for analytical purposes, a Log_10_ transformation was applied to the computed results, following the calculation approaches employed in TYG, AIP, and similar indices.

### Univariate and Multivariate Analyses

To identify the independent influencing factors of the outcome variables, univariate and multivariate logistic regression analyses, supplemented by random forest models to present the analytical results, were conducted. The logistic regression analyses and result organization were performed using the “rms” R package,^[^ ^35^ ^]^ following methodologies referenced in prior studies.^[^ ^36^ ^]^ Briefly, logistic regression functions for model selection was employed. The dataset was split into training (70%) and testing sets. A fivefold cross‐validation approach was utilized, with the maximum number of iterations set to 1000 for model training. After cross‐validation, the optimal number of iterations for logistic regression selection was determined to be 20, while other parameters were maintained at their default values. The analytical results were organized using the “autoReg” and “rrtable” packages.^[^ ^37^ ^]^ ROC curves were plotted using the “pROC” package.^[^ ^38^ ^]^ and Decision Curve Analysis (DCA) curves were generated with the “rmda” package.^[^ ^39^ ^]^ Finally, the results of univariate and multivariate logistic regression analyses were visualized as forest plots using the “forestploter” package.^[^ ^40^ ^]^

### Restricted Cubic Splines Analysis

In this study, RCS analysis was primarily employed to investigate the linear/nonlinear relationships and risk thresholds between GLM7 and various diseases. The “rms” and “ggrcs” packages^[^ ^35^, ^41^ ^]^ were utilized to plot RCS curves, while the “ggplot2”, “scales”, and “cowplot” packages were employed for figure refinement and visualization.^[^ ^42^ ^]^ The core code was adapted from tutorials provided by each respective package. Specifically, the lrm function was used to fit binary logistic regression models, and the rcs function was applied to model the continuous variable GLM7 using restricted cubic splines with 5 knots specified. This approach divided the distribution of GLM7 into multiple intervals, within which polynomial functions were fitted to flexibly capture nonlinear relationships between GLM7 and various diseases. Additionally, the “segmented” package^[^ ^43^ ^]^ was employed to fit piecewise logistic regression models, analyzing the nonlinear relationships between the continuous variable GLM7 and binary disease outcomes. The package automatically estimated breakpoints, which were interpreted as risk thresholds.

### Machine Learning Model

In this investigation, the previously established XGBoost machine learning algorithm for the construction of predictive models was employed.^[^ ^44^ ^]^ Briefly, the dataset was randomly partitioned into training and test subsets at a 0.7:0.3 ratio. For hyperparameter tuning, the R package “caret”^[^ ^45^ ^]^ was employed, using fivefold cross‐validation for parameter tuning, initiating the process with a grid search encompassing 200 iterations, a maximum tree depth of 6, a learning rate (η) of 0.1, a minimum loss reduction threshold of 0.1 for node splitting, a subsampling ratio of 80% for features, a minimum child node weight of 3, and a subsampling ratio of 80% for observations, along with additional tuning parameters. After optimization, performance metrics (e.g., accuracy and precision) were computed for both training and evaluation datasets. ElasticNet regression exhibited superior performance in terms of the area under the curve (AUC) on cross‐validated training data. The receiver operating characteristic (ROC) curve was visualized using the R package “pROC,”^[^ ^38^ ^]^ and the precision‐recall curve (PRC) was computed and plotted via the “PRROC” package.^[^ ^46^ ^]^ A confusion matrix was generated using the R package “ggplot2.” All data processing and statistical analyses were conducted within RStudio (2025.05.0‐496) using R version 4.4.3.

### Statistical Analysis

The statistical examination of data sourced from NHANES and CHARLS was carried out in strict compliance with their corresponding protocols, integrating sample weights (WTMEC2YR), incorporating stratification, and adjusting for clustering effect to address the intricacies of the survey designs. Survey‐weighted linear regression was employed to evaluate the temporal trends in age‐standardized outcomes. Subsequently, a repeated analysis to approximate the prevalence of risk factors for each disease, categorizing them by age, race, gender, educational background, marital status, alcohol consumption patterns, smoking habits, and physical activity levels, was conducted. Thereafter, complete‐case analyses were undertaken based on the study outcomes. Continuous variables that followed a normal distribution were articulated as means ± standard deviation (SD), whereas those deviating from normality were reported as medians. Categorical variables were delineated in terms of frequencies and percentages. The χ^2^ test was administered for categorical variables, ANOVA for normally distributed continuous variables, and the Kruskal–Wallis test for nonparametric data. All statistical tests employed a two‐tailed approach, and statistical significance was defined as *p* < 0.05. The “survey” package^[^ ^47^ ^]^ was utilized for executing the statistical analyses, and the “autoReg” package (available at: https://github.com/cardiomoon/autoReg) was harnessed for summarizing the results. To appraise the comprehensive influence of various factors on each disease, receiver operating characteristic (ROC) curves were constructed to depict the impact of individual variables on disease outcomes, utilizing the area under the curve (AUC) as a quantitative metric for enhancement. Logistic regression models were employed to scrutinize the associations between variables and outcome risks, with odds ratios (OR) and 95% confidence intervals computed and results visualized through forest plots. Furthermore, the relationship between GLM7 and outcomes was explored, and RCS analysis was applied to elucidate the dose‐response relationship with outcome risks. Ultimately, machine learning techniques grounded in the XGBoost package were leveraged for constructing predictive models.^[^ ^48^ ^]^ All data manipulation and statistical computations were executed using R (version 4.4.3).

### Ethics Approval and Consent to Participate

The NHANES protocol was reviewed and approved by the Institutional Review Board of the National Center for Health Statistics. Before data collection commenced, all participants enrolled in the study provided written informed consent. Similarly, the CHARLS study received ethical approval from Peking University's Institutional Review Board (IRB00001052‐11015), and written informed consent was obtained from all participants prior to their involvement in the research.

## Conflict of Interest

The authors declare no conflict of interest.

## Author Contributions

X.C. performed the data analysis and drafted the manuscript. X.F., S.C., and W.Z. were responsible for data acquisition and pre‐processing. Z.W. and S.X. collaborated on the study design. Z.W., Y.D., and S.X. contributed to editing and proofreading of the article. P.C.E. and H.S. proofread the manuscript and offer significant conceptual comments for the manuscript. S.X., Y.D., and J.W. conceived the research, supervised its execution, and revised the manuscript.

## Supporting information



Supporting Information

Supporting Information

## Data Availability

The data that support the findings of this study are available in the supplementary material of this article.
