# Supplementary material for: GLM7 – A Novel Composite Glycolipid Index Derived from Routine Health Indicators for Enhanced Diagnosis and Prediction of Multimorbidity
Source: Adv Sci (Weinh). 2025 Aug 28;12(42):e10552. doi: 10.1002/advs.202510552 (PMC12622479; doi:10.1002/advs.202510552)
Supplement: Supplementary file 1 — Supporting Information [file ADVS-12-e10552-s001.docx]

**Supporting Information**

**GLM7 - A Novel Composite Glycolipid Index Derived from Routine Health Indicators for Enhanced Diagnosis and Prediction of Multimorbidity**

Zhihua Wang^1^, Shuo Chen^1^, Xiaojun Feng^1^, Xi Chen^1^, Paul C Evans^3^, Hans Strijdom^4^, Yu Ding^1,2^*, Jianping Weng^1,2^*, Suowen Xu^1,2^*

1. Department of Endocrinology, Centre for Leading Medicine and Advanced Technologies of IHM, The First Affiliated Hospital of USTC, Division of Life Sciences and Medicine, University of Science and Technology of China, Hefei, 230001, China

2. Anhui Provincial Key Laboratory of Metabolic Health and Panvascular Diseases, Hefei, 230001, China.

3. William Harvey Research Institute, Barts and The London Faculty of Medicine and Dentistry, Queen Mary University of London, London, EC1M 6BQ, United Kingdom.

4. Centre for Cardio-metabolic Research in Africa, Division of Medical Physiology, Faculty of Medicine and Health Sciences, Stellenbosch University, Cape Town, 8000, South Africa.

*Correspondence: Jianping Weng ([wengjp@ustc.edu.cn](mailto:wengjp@ustc.edu.cn)), Suowen Xu ([sxu1984@ustc.edu.cn](mailto:sxu1984@ustc.edu.cn)), Yu Ding (yuding6815@163.com)

**Content of Supporting Information**

**1. Figure S1**. SHAP value-based interpretation of factor weights in the GLM7 prediction model for different disease outcomes.

2. **Figure S2**. Comparative analysis of diagnostic or predictive performance between the GLM7 index and established indices (TYG, TYG_BMI, and AIP) for disease outcomes.

1. **Table S1**. List of all data sources.
2. **Table S2**. Univariate regression analysis of 49 factors on cardiovascular diseases.
3. **Table S3**. Univariate regression analysis of 49 factors on diabetes mellitus.
4. **Table S4**. Univariate regression analysis of 49 factors on liver diseases.
5. **Table S5**. Univariate regression analysis of 49 factors on cancers.
6. **Table S6.** Univariate regression analysis of 49 factors on comorbidities.
7. **Table S7.** Overall trends of 7 routine indicators and the relationship between GLM7 and disease.

**
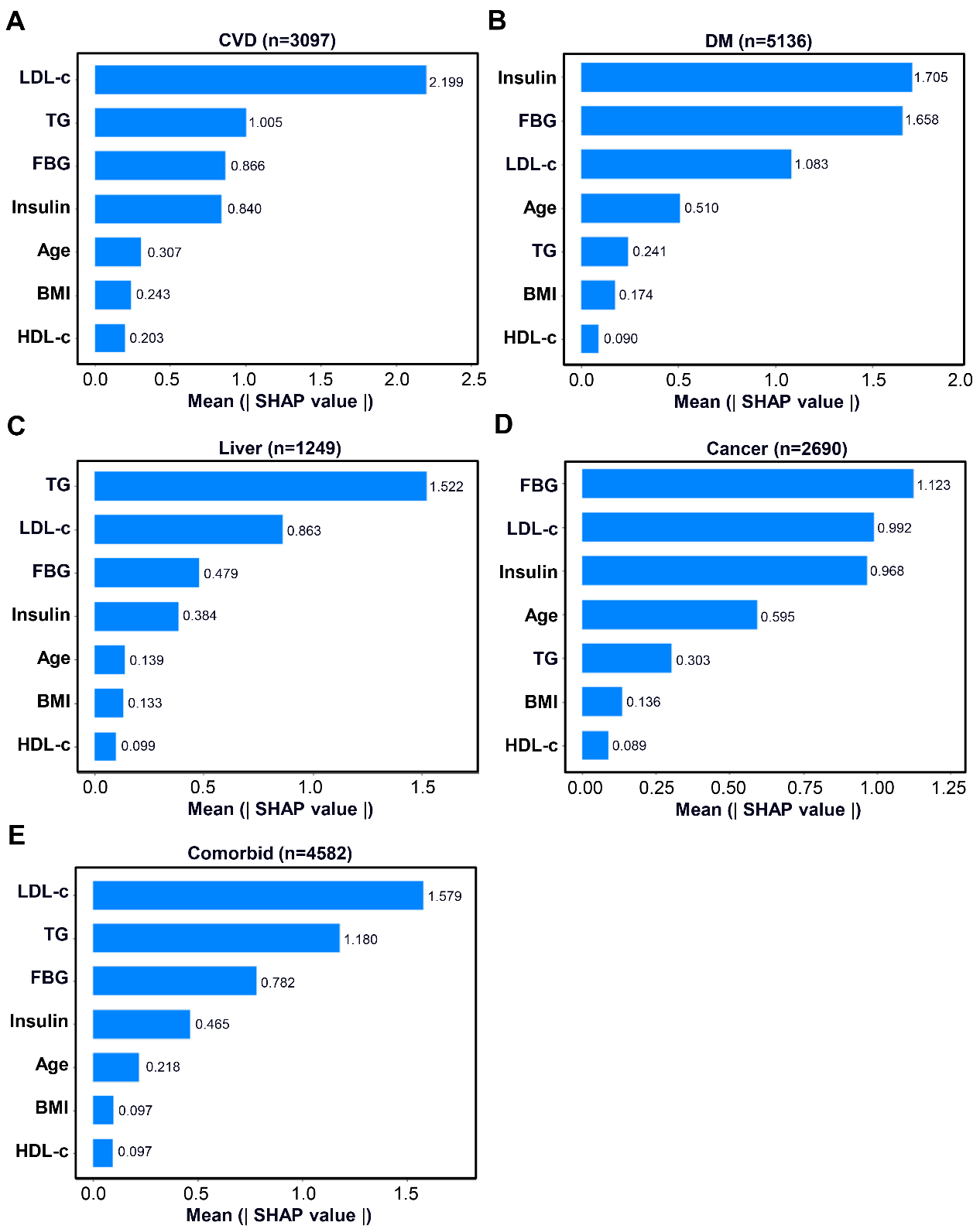
**

**Figure S1. SHAP value-based interpretation of factor weights in the GLM7 prediction model for different disease outcomes. (A)** Bar plot illustrating the SHAP values of seven factors in the GLM7 model for predicting cardiovascular disease. **(B)** Bar plot illustrating the SHAP values of seven factors in the GLM7 model for predicting diabetes mellitus. **(C)** Bar plot illustrating the SHAP values of seven factors in the GLM7 model for predicting liver diseases. **(D)** Bar plot illustrating the SHAP values of seven factors in the GLM7 model for predicting cancer. **(E)** Bar plot illustrating the SHAP values of seven factors in the GLM7 model for predicting comorbidities.

**
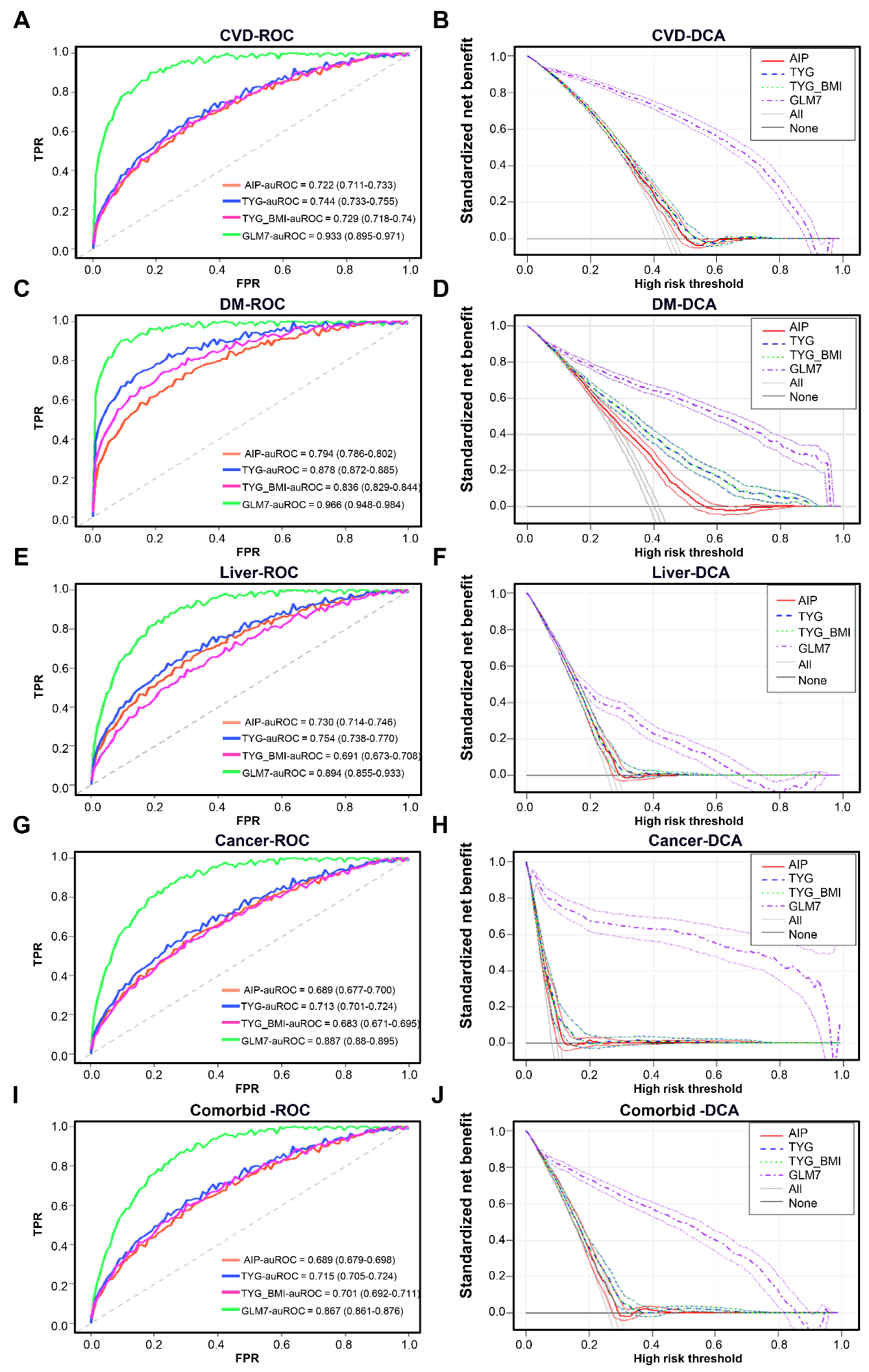
**

**Figure S2. Comparative analysis of diagnostic or predictive performance between the GLM7 index and established indices (TYG, TYG_BMI, and AIP) for disease outcomes. (A)** ROC analysis of the four indices for cardiovascular disease. **(B)** DCA analysis of the four indices for cardiovascular disease. **(C)** ROC analysis of the four indices for diabetes mellitus. **(D)** DCA analysis of the four indices for diabetes mellitus. **(E)** ROC analysis of the four indices for liver diseases. **(F)** DCA analysis of the four indices for liver diseases. **(G)** ROC analysis of the four indices for cancer. **(H)** DCA analysis of the four indices for cancer. **(I)** ROC analysis of the four indices for comorbidities. **(J)** DCA analysis of the four indices for comorbidities.
